# Psychosocial Work Environment in the Operating Room of Greek Public Hospitals: A Cross-Sectional Study Among Ophthalmology Residents Using the Copenhagen Psychosocial Questionnaire

**DOI:** 10.7759/cureus.95909

**Published:** 2025-11-01

**Authors:** Vasiliki Dimaki, Alexandra Mpeza, Nikolaos Kappos, Antonia Vlachou, Dimitrios Papaconstantinou, Klio I Chatzistefanou, Marilita M Moschos

**Affiliations:** 1 Department of Health Promotion and Education, Medical School, National and Kapodistrian University of Athens, Athens, GRC; 2 Department of Philology, National and Kapodistrian University of Athens, Athens, GRC; 3 Department of Ophthalmology, Athens Naval Hospital, Athens, GRC; 4 Department of Nursing, General Hospital of Athens, Athens, GRC; 5 Department of Ophthalmology, Medical School, National and Kapodistrian University of Athens, Athens, GRC; 6 First Department of Ophthalmology, Medical School, National and Kapodistrian University of Athens, Athens, GRC

**Keywords:** copsoq ii, greece, job satisfaction, ophthalmology residents, psychosocial work environment, residency training

## Abstract

Background: The psychosocial work environment influences medical residents' well-being, job satisfaction, and performance. In surgical specialties such as ophthalmology, residents face unique demands due to high workloads, technical complexity, and hierarchical structures. However, national-level evidence from Greece is scarce, limited to targeted interventions.

Methods: A quantitative, cross-sectional study was conducted among 81 ophthalmology residents in public hospitals in Athens, Greece, using the validated Greek version of the Copenhagen Psychosocial Questionnaire, second version (COPSOQ II). Sociodemographic characteristics were collected, and correlations between psychosocial factors and job satisfaction were analyzed using Statistical Package for the Social Sciences version 26.0 (IBM Corp., Armonk, NY).

Results: The response rate was 100%. Higher quantitative and cognitive demands were associated with increased emotional demands (ρ = 0.28, p = 0.012; ρ = 0.40, p < 0.001), emotional suppression (ρ = 0.23, p = 0.036; ρ = 0.26, p = 0.021), and sensory strain (ρ = 0.42, p < 0.001), along with reduced freedom in work (ρ = -0.24, p = 0.033), meaning of work (ρ = -0.22, p = 0.047), social support (ρ = -0.26, p = 0.019), and job satisfaction (ρ = -0.24, p = 0.029). Job resources, such as influence at work, possibilities for development, role clarity, and social support, were positively related to job satisfaction (ρ = 0.26-0.53, p < 0.05) and team feeling (ρ = 0.26-0.45, p < 0.01) and negatively related to role conflicts (ρ = -0.23 to -0.47, p < 0.05). Role conflicts were strongly associated with lower leadership quality (ρ = -0.36, p = 0.001), weaker social support (ρ = -0.47, p < 0.001), and reduced job satisfaction (ρ = -0.43, p < 0.001). Development opportunities showed a particularly strong positive association with job satisfaction (ρ = 0.26, p = 0.020) and meaning of work (ρ = 0.54, p < 0.001).

Conclusions: These findings highlight the importance of addressing psychosocial stressors in residency programs. Interventions such as workload management, mentoring, and leadership training could foster healthier work environments, enhance job satisfaction, and ultimately improve patient care.

## Introduction

The operating room (OR) is widely recognized as one of the most challenging environments for medical professionals. In surgical specialties such as ophthalmology, residents must not only acquire complex technical skills but also develop nontechnical skills such as teamwork, communication, situational awareness, and decision-making under stress [1]. These demands are further intensified by the hierarchical structure of the OR, high patient turnover, limited time for supervision, and a culture of zero tolerance for error [2].

The psychosocial work environment refers to the interaction between individuals and their working conditions, encompassing factors such as emotional demands, job insecurity, social support, autonomy, and work-life balance [3,4]. Numerous studies have demonstrated that a negative psychosocial work environment is associated with burnout, depression, anxiety, and reduced work performance among healthcare professionals [5-7].

Ophthalmology residency programs pose unique psychosocial challenges. The complexity of microsurgical procedures, the pressure to maintain precision, and the limited opportunities for hands-on training in high-volume centers contribute to heightened levels of occupational stress [8]. Furthermore, residents often experience role ambiguity, limited decision-making authority, and a lack of formal support structures, which can negatively affect both their learning and well-being [9].

In Greece, the economic crisis and the COVID-19 pandemic have placed additional pressure on public hospitals and their staff. Residents in ophthalmology, particularly in public hospital surgical departments, have faced increased workloads, limited resources, and emotional exhaustion [10,11]. However, there is a notable lack of published research exploring the psychosocial work environment of ophthalmology residents in Greece, particularly in the context of the OR.

The aim of this study was to assess the psychosocial work environment of ophthalmology residents working in surgical departments of public hospitals in Athens, Greece. Specifically, the research aimed to (a) identify key psychosocial factors related to residents’ work experience in the OR, (b) examine associations between these factors and demographic/professional characteristics, and (c) highlight areas that could inform targeted interventions to improve resident well-being and training quality. The Copenhagen Psychosocial Questionnaire (COPSOQ II), validated in Greek by Zigkiri et al. [12], was selected to examine multiple dimensions of the psychosocial work environment. By identifying both stressors and protective factors, this study seeks to contribute to the development of more supportive training environments that promote resident well-being, clinical competence, and, ultimately, better patient care.

## Materials and methods

This was a quantitative, cross-sectional study conducted to investigate the psychosocial work environment of ophthalmology residents in the OR setting. The Greek version of the COPSOQ II was used as the main instrument, validated in Greek by Zigkiri et al. [12]. As COPSOQ is freely available for academic use, no additional permission was required beyond proper citation. This questionnaire evaluates job demands, job resources, and outcomes. Items were rated on a 5-point Likert scale, with higher scores indicating greater intensity of each dimension (see the Appendix). Reliability coefficients (Cronbach’s alpha/KR-20) for the subscales used in this study are presented in Table 2.

It was calculated that with the sample size of at least 73 participants, the study will have 90% power to perform a linear regression analysis with the COPSOQ II subscales as dependent variables at a significance level of 0.05 and for effect sizes equal to or greater than 0.15.

Participants included 81 ophthalmology residents from seven public hospitals in the Athens metropolitan area. The inclusion criteria were 1) current residency training in ophthalmology, 2) active participation in surgical procedures as part of their training, and 3) voluntary willingness to participate. Additional sociodemographic data (age, gender, relationship status, year of residency, etc.) were also collected.

Data were gathered between March 2021 and September 2021. Questionnaires were given to all ophthalmology residents in the seven participating public hospitals in the Athens metropolitan area, along with sealed envelopes to ensure anonymity, and all were returned completed. The research, conducted during the COVID-19 pandemic, fully complied with the established safety and hygiene protocols.

A collection period of one to two weeks was allowed, after which the sealed envelopes were collected by the researchers. All responses were manually entered into a digital form (Google Forms; Google LLC, Mountain View, CA) created by one of the researchers and subsequently exported to Excel format (Microsoft Corporation, Redmond, WA). Data accuracy was ensured through double-checking and cross-validation of entries by the research team to minimize potential errors and maintain data integrity. Descriptive and inferential statistical analyses were performed using Statistical Package for the Social Sciences (SPSS) version 26.0 (IBM Corp., Armonk, NY), including correlation tests between key psychosocial factors and demographic characteristics.

This study received ethical approval from the Scientific Council of the General State Hospital of Athens "G. Gennimatas" (IRB number: 25724; approval date: September 28, 2021), which served as the coordinating center. Given the observational, anonymous, and non-interventional nature of the study, no additional formal approvals were required.

Quantitative variables were expressed as mean (standard deviation (SD)) or as median (interquartile range). Categorical variables were expressed as absolute and relative frequencies. Spearman’s correlation coefficients were used to explore the association of two continuous variables. Multiple linear regression analyses were used with the dependent COPSOQ ΙΙ subscales in a stepwise method (p for entry = 0.05, p for removal = 0.10). This method allows the identification of the most significant independent predictors among correlated psychosocial variables while accounting for multicollinearity. Adjusted regression coefficients (β) with standard errors were computed from the results of the linear regression analyses. Internal consistency reliability was determined by the calculation of Cronbach’s alpha coefficient or KR-20 coefficient. Scales with reliabilities equal to or greater than 0.70 were considered acceptable. All reported p values are two-tailed. Statistical significance was set at p < 0.05, and analyses were conducted using SPSS statistical software (version 26.0).

## Results

A total of 81 ophthalmology residents from public hospitals in Athens participated in the study (response rate: 100%). Of the respondents, 43.2% were men (n = 35) and 56.8% were women (n = 46), with a mean age of 35 years (SD = 4.6). Most participants were in a relationship (46.9%) or single (32.1%), whereas 21% were married. The mean duration of ophthalmology training was 2.6 years (SD = 1.5), and almost all participants (98.8%) were employed full-time. Nearly half (44.4%) reported frequent night shifts, reflecting the demanding nature of the residency training programs. A notable proportion held postgraduate degrees (19.8% MSc and 9.9% PhD), indicating a high educational level among participants. These characteristics are summarized in Table 1.

Regarding psychosocial characteristics, Table 2 presents the descriptive statistics and reliability indexes (Cronbach’s alpha or KR-20) for each subscale of the COPSOQ II questionnaire. The highest mean scores were observed in the subscales of sensory demands (M = 91.67, SD = 11.22), meaning of work (M = 80.76, SD = 15.68), and job satisfaction (M = 72.22, SD = 14.22). These findings suggest that, despite the high attentional demands of ophthalmic surgery, most residents perceived their work as meaningful and generally satisfying. Conversely, lower mean scores were recorded in job insecurity (M = 26.23, SD = 29.82), demands for hiding emotions (M = 42.28, SD = 19.62), and emotional demands (M = 45.06, SD = 19.93), revealing potential sources of strain related to uncertainty and emotional regulation. All COPSOQ II subscales demonstrated acceptable to excellent internal consistency, with Cronbach’s alpha values ranging from 0.70 to 0.92. The job insecurity subscale was assessed using the KR-20 reliability index, which also showed good reliability (KR-20 = 0.75).

Table 3 displays correlation coefficients between key psychosocial factors assessed via the COPSOQ II. In this section, we emphasize reporting the most substantial and statistically significant associations that relate to the psychosocial work environment and well-being of ophthalmology residents.

Quantitative demands demonstrated positive associations with emotional demands (ρ = 0.28; p = 0.012) and demands for hiding emotions (ρ = 0.23; p = 0.036), indicating that higher workload was accompanied by greater emotional strain. In contrast, quantitative demands were negatively correlated with job satisfaction (ρ = -0.24; p = 0.029), freedom in work (ρ = -0.24; p = 0.033), and team feeling (ρ = -0.29; p = 0.008), suggesting that excessive workload may undermine autonomy and collaboration. Cognitive demands, however, correlated positively with possibilities for development (ρ = 0.50; p < 0.001), implying that intellectually challenging tasks might also promote professional growth.

**Table 1:** Demographic and professional characteristics of the participants (n = 81) Values are presented as frequencies (n) and percentages. Age and duration of residency are presented as mean (SD)

Characteristics	Value
Gender, n (%)	
Male	35 (43.2)
Female	46 (56.8)
Relationship status, n (%)	
Married	17 (21.0)
Single	26 (32.1)
In a relationship	38 (46.9)
Employment status, n (%)	
Full-time	80 (98.8)
Part-time	1 (1.2)
Night shifts, n (%)	
Frequently	36 (44.4)
Not frequently	45 (55.6)
Education level, n (%)	
MSc	16 (19.8)
PhD	8 (9.9)
Age (years), mean (SD)	35.0 (4.6)
Duration of residency (years), mean (SD)	2.6 (1.5)

**Table 2:** Descriptive measures and reliability indexes of COPSOQ II subscales Values are presented as minimum, maximum, mean (SD), and median (IQR). Internal consistency was assessed with Cronbach’s alpha; for job insecurity, the KR-20 index was used. Scales with reliabilities ≥0.70 were considered acceptable IQR: interquartile range; SD: standard deviation; COPSOQ II: Copenhagen Psychosocial Questionnaire II

Subscales	Minimum	Maximum	Mean (SD)	Median (IQR)	Cronbach’s alpha
Quantitative demands	20.83	79.17	47.84 (12.73)	45.83 (41.67-54.17)	0.70
Cognitive demands	31.25	100.00	70.52 (15.12)	68.75 (62.50-81.25)	0.70
Emotional demands	0.00	91.67	45.06 (19.93)	41.67 (33.33-58.33)	0.70
Demands for hiding emotions	0.00	87.50	42.28 (19.62)	37.50 (25.00-62.50)	0.75
Sensory demands	62.50	100.00	91.67 (11.22)	100.00 (87.50-100.00)	0.83
Influence at work	8.33	91.67	49.69 (19.07)	41.67 (41.67-66.67)	0.71
Possibilities for development	37.50	100.00	71.76 (14.86)	68.75 (62.50-81.25)	0.76
Freedom in work	0.00	93.75	51.39 (21.31)	50.00 (37.50-68.75)	0.81
Meaning of work	33.33	100.00	80.76 (15.68)	75.00 (66.67-100.00)	0.82
Commitment to the work environment	12.50	93.75	55.63 (16.76)	56.25 (43.75-68.75)	0.72
Predictability	0.00	100.00	59.26 (19.64)	62.50 (50.00-75.00)	0.83
Role clarity	18.75	100.00	68.98 (15.39)	75.00 (62.50-75.00)	0.85
Role conflicts	0.00	93.75	43.67 (20.58)	43.75 (25.00-56.25)	0.77
Quality of leadership	0.00	100.00	60.49 (24.74)	62.50 (43.75-75.00)	0.91
Social support	18.75	100.00	66.90 (19.70)	68.75 (56.25-81.25)	0.84
Support in the workplace	12.50	87.50	55.40 (16.99)	62.50 (50.00-62.50)	0.79
Social relationships	25.00	100.00	72.22 (18.54)	75.00 (62.50-87.50)	0.75
Team feeling	25.00	100.00	69.55 (17.98)	75.00 (66.67-75.00)	0.92
Job insecurity	0.00	100.00	26.23 (29.82)	25.00 (0.00-50.00)	0.75+
Job satisfaction	25.00	100.00	72.22 (14.22)	75.00 (68.75-75.00)	0.90

**Table 3:** Spearman's correlation coefficients (ρ) among COPSOQ II subscales ^*^p < 0.05; ^**^p < 0.01; ^***^p < 0.001 CD: cognitive demands; CL: role clarity; CO: role conflicts; CW: commitment to the work environment; ED: emotional demands; FW: freedom in work; HE: demands for hiding emotions; IN: influence at work; JIN: job insecurity; JS: job satisfaction; MW: meaning of work; PD: possibilities for development; PR: predictability; QD: quantitative demands; QL: quality of leadership; SED: sensory demands; SR: social relationships; SS: social support; SW: support in workplace; TF: team feeling; COPSOQ II: Copenhagen Psychosocial Questionnaire II

Parameter	QD	CD	ED	HE	SED	IN	PD	FW	MW	CW	PR	CL	CO	QL	SS	SW	SR	TF	JIN	JS
QD	1.00	0.21	0.28^*^	0.23^*^	0.19	0.01	0.02	-0.24^*^	-0.22^*^	-0.17	-0.19	-0.18	0.13	-0.19	-0.26*	-0.10	-0.15	-0.29^**^	0.15	-0.24^*^
CD	0.21	1.00	0.40^***^	0.26^*^	0.42^***^	0.40^***^	0.53^***^	0.03	0.13	0.13	0.08	0.16	0.20	0.16	0.06	0.11	0.06	0.12	-0.13	-0.02
ED	0.28^*^	0.40^***^	1.00	0.44^***^	0.09	0.27^*^	0.19	-0.04	-0.23^*^	0.06	-0.07	-0.02	0.19	0.01	-0.01	-0.05	0.11	-0.11	0.14	-0.20
HE	0.23^*^	0.26^*^	0.44^***^	1.00	0.05	0.02	0.05	-0.25^*^	-0.20	0.01	-0.22^*^	-0.17	0.36^**^	-0.27^*^	-0.46^***^	-0.21	-0.21	-0.30^**^	0.22^*^	-0.19
SED	0.19	0.42^***^	0.09	0.05	1.00	0.13	0.44^***^	-0.07	0.41^***^	0.10	0.17	0.40^***^	-0.02	0.09	0.07	-0.01	-0.01	0.11	-0.10	0.08
IN	0.01	0.40^***^	0.27^*^	0.02	0.13	1.00	0.58^***^	0.07	0.24^*^	0.15	0.09	0.20	-0.02	0.18	0.21	0.25^*^	0.02	0.17	-0.07	0.03
PD	0.02	0.53^***^	0.19	0.05	0.44^***^	0.58^***^	1.00	0.18	0.54^***^	0.42^***^	0.29^**^	0.35^**^	0.01	0.34^**^	0.18	0.28^*^	0.09	0.32^**^	0.00	0.26^*^
FW	-0.24^*^	0.03	-0.04	-0.25^*^	-0.07	0.07	0.18	1.00	0.15	0.33^**^	0.38^**^	0.30^**^	-0.15	0.30^**^	0.39^***^	0.27^*^	0.30^**^	0.26^*^	0.00	0.38^***^
MW	-0.22^*^	0.13	-0.23^*^	-0.20	0.41^***^	0.24^*^	0.54^***^	0.15	1.00	0.53^***^	0.37^**^	0.53^***^	-0.47^***^	0.39^***^	0.36^**^	0.44^***^	0.10	0.45^***^	-0.17	0.53^***^
CW	-0.17	0.13	0.06	0.01	0.10	0.15	0.42^***^	0.33^**^	0.53^***^	1.00	0.56^***^	0.48^***^	-0.15	0.51^***^	0.29^*^	0.55^***^	0.03	0.41^***^	0.13	0.47^***^
PR	-0.19	0.08	-0.07	-0.22^*^	0.17	0.09	0.29^**^	0.38^**^	0.37^**^	0.56^***^	1.00	0.55^***^	-0.30^**^	0.45^***^	0.35^**^	0.42^***^	0.09	0.42^***^	-0.01	0.45^***^
CL	-0.18	0.16	-0.02	-0.17	0.40^***^	0.20	0.35^**^	0.30^**^	0.53^***^	0.48^***^	0.55^***^	1.00	-0.23^*^	0.44^***^	0.43^***^	0.42^***^	0.14	0.45^***^	-0.01	0.40^***^
CO	0.13	0.20	0.19	0.36^**^	-0.02	-0.02	0.01	-0.15	-0.47^***^	-0.15	-0.30^**^	-0.23^*^	1.00	-0.36^**^	-0.47^***^	-0.22	-0.06	-0.29^**^	0.06	-0.43^***^
QL	-0.19	0.16	0.01	-0.27^*^	0.09	0.18	0.34^**^	0.30^**^	0.39^***^	0.51^***^	0.45^***^	0.44^***^	-0.36^**^	1.00	0.65^***^	0.55^***^	0.19	0.56^***^	0.00	0.43^***^
SS	-0.26^*^	0.06	-0.01	-0.46^***^	0.07	0.21	0.18	0.39^***^	0.36^**^	0.29^*^	0.35^**^	0.43^***^	-0.47^***^	0.65^***^	1.00	0.50^***^	0.41^***^	0.66^***^	0.02	0.35^**^
SW	-0.10	0.11	-0.05	-0.21	-0.01	0.25^*^	0.28^*^	0.27^*^	0.44^***^	0.55^***^	0.42^***^	0.42^***^	-0.22	0.55^***^	0.50^***^	1.00	0.21	0.45^***^	0.00	0.23^*^
SR	-0.15	0.06	0.11	-0.21	-0.01	0.02	0.09	0.30^**^	0.10	0.03	0.09	0.14	-0.06	0.19	0.41^***^	0.21	1.00	0.34^**^	0.02	0.02
TF	-0.29^**^	0.12	-0.11	-0.30^**^	0.11	0.17	0.32^**^	0.26^*^	0.45^***^	0.41^***^	0.42^***^	0.45^***^	-0.29^**^	0.56^***^	0.66^***^	0.45^***^	0.34^**^	1.00	-0.09	0.48^***^
JIN	0.15	-0.13	0.14	0.22^*^	-0.10	-0.07	0.00	0.00	-0.17	0.13	-0.01	-0.01	0.06	0.00	0.02	0.00	0.02	-0.09	1.00	-0.16
JS	-0.24^*^	-0.02	-0.20	-0.19	0.08	0.03	0.26^*^	0.38^***^	0.53^***^	0.47^***^	0.45^***^	0.40^***^	-0.43^***^	0.43^***^	0.35^**^	0.23^*^	0.02	0.48^***^	-0.16	1.00

**Table 4:** Multivariate linear regression results with COPSOQ subscales as dependent variables, in a stepwise method Regression coefficients (β), SEs, and p values are reported. p < 0.05 was considered statistically significant Empty rows indicate that no independent variables were found to be significantly associated with these dependent variables SE: standard error; COPSOQ: Copenhagen Psychosocial Questionnaire

Dependent variable	Independent variables	β+	SE++	P
Quantitative demands	Years in specialty	0.02	0.01	0.01
	Family status (single vs. married/in a relationship)	0.07	0.03	0.02
Cognitive demands	Years in specialty	0.03	0.01	0.00
Emotional demands	Gender (men vs. women)	-0.02	0.01	0.00
Demands for hiding emotions	-	-	-	-
Sensory demands	Years in specialty	0.01	0.00	0.02
	Night shifts (yes, often vs. sometimes)	0.03	0.01	0.04
Influence at work	Years in specialty	0.03	0.01	0.03
	Family status (single vs. married/in a relationship)	-0.09	0.04	0.04
Possibilities for development	-	-	-	-
Freedom in work	-	-	-	-
Meaning of work	Gender (men vs. women)	0.05	0.02	0.01
Commitment to the work environment	Gender (men vs. women)	0.08	0.04	0.04
Predictability	Gender (men vs. women)	0.11	0.06	0.05
Role clarity	Gender (men vs. women)	0.06	0.03	0.03
	Age	0.01	0.00	0.02
Role conflicts	-	-	-	-
Quality of leadership	-	-	-	-
Social support	-	-	-	-
Support in the workplace	Gender (men vs. women)	0.08	0.04	0.03
Social relationships	MSc and/or PhD holder (yes vs. no)	-0.07	0.03	0.03
Team feeling	-	-	-	-
Job insecurity	Family status (single vs. married/in a relationship)	0.55	0.18	0.00
	Night shifts (yes, often vs. sometimes)	0.42	0.17	0.02
	MSc and/or PhD holder (yes vs. no)	-0.45	0.19	0.02
Job satisfaction	Medical school graduate (in Greece vs. abroad)	-0.06	0.03	0.03

Similarly, the meaning of work correlated positively with job satisfaction (ρ = 0.53; p < 0.001) and team feeling (ρ = 0.45; p < 0.001), highlighting the motivational value of purposeful work. Conversely, role conflicts showed negative correlations with social support (ρ = -0.47; p = 0.008) and job satisfaction (ρ = -0.43; p < 0.001), illustrating how ambiguous or overlapping duties can erode morale and cooperation. Furthermore, support in the workplace, quality of leadership, and team feeling all demonstrated strong positive correlations with job satisfaction, indicating that a well-organized, supportive, and collaborative environment may enhance residents’ well-being and perceived training quality.

Multivariate linear regression analysis, shown in Table 4, indicated that years in specialty were significantly associated with higher quantitative and cognitive demands, as well as with influence at work. Male residents reported fewer emotional demands but higher role clarity and workplace support, while single participants and those working frequent night shifts experienced heightened job insecurity. In contrast, residents with an MSc or PhD degree reported reduced levels of insecurity, possibly due to stronger professional confidence. Additionally, job satisfaction was significantly lower among graduates from Greek medical schools.

## Discussion

This study examined the psychosocial work environment of ophthalmology residents in Greek public hospitals, focusing on the relationships between job demands, job resources, and job satisfaction. The analysis in this research revealed that higher quantitative and cognitive demands were associated with greater emotional demands and a need for emotional suppression, while job resources, such as influence at work, possibilities for development, social support, and team feeling, were positively related to job satisfaction and negatively related to role conflicts. Such national-level evidence is scarce, and these results, therefore, provide a rare snapshot of the working lives and psychosocial environment of medical residents in Greece in the OR.

More specifically, high quantitative demands were linked not only to emotional strain and suppression but also to decreased freedom in work, meaning in work, social support, team feeling, and job satisfaction. They were also positively associated with demands for hiding emotions, a finding that may reflect the emotional regulation required in high-pressure surgical settings. This pattern aligns with the Job Demands-Resources model and previous findings on the toll that workload can take on well-being [13]. In the context of the OR, these high demands may require residents to manage their emotions carefully while working under pressure, which can subtly erode perceived support and team feeling [14].

Moreover, cognitive demands were associated with a dual impact: on one hand, they were linked to increased emotional demands, sensory strain, and demands for hiding emotions; on the other hand, they were correlated with greater influence at work and more opportunities for professional growth. This pattern reflects the demanding yet developmental nature of surgical training, where intense mental workload coexists with the potential for skill enhancement. However, sustained emotional strain appeared to diminish the perceived meaning of work, underscoring the risk that prolonged psychological pressure may erode intrinsic motivation [15].

Considering the impact of heavy workload, complex decision-making requirements, and high emotional demands on medical residents’ well-being, role conflicts emerged as one of the most damaging factors, associated with lower leadership quality, social support, team feeling, and job satisfaction. On the other hand, role conflicts were also positively associated with demands for hiding emotions, suggesting that unclear responsibilities may increase the pressure to mask emotions in the OR. Clearer role definitions and better communication within surgical teams could mitigate these effects [16]. In Greece, where residency training is almost exclusively in public hospitals, the combination of high patient volumes, understaffing, and limited resources likely amplifies both the demands and the conflicts.

Cultural factors may also shape these dynamics of high job demands, limited work resources, and role conflict, as well as their impact on team feeling and job satisfaction. In Greece, family support often serves as a vital buffer against workplace stress. In the present study, single residents showed significantly lower scores of social support and team feeling compared with their married participants, suggesting that the absence of close family ties in daily life may heighten reliance on workplace connections for emotional support. This finding aligns with the cultural importance of family bonds in Greece, where close personal relationships often act as a protective factor against occupational stress [17].

Job insecurity in this study was significantly associated with lower perceptions of social support and team feeling, indicating that concerns about career stability may erode collaborative relationships in the OR. This finding aligns with international literature, indicating that perceived instability in future career prospects can negatively affect motivation and workplace relationships among medical residents [18].

Furthermore, findings from the multiple linear regression analysis (Table 4) further highlighted notable demographic and professional differences among the residents. Demographic and professional factors appeared to shape how residents perceived their psychosocial work environment. Gender differences were evident, with men generally reporting lower emotional demands and greater clarity, support, and autonomy at work, whereas women were often faced with more emotional strain within the surgical setting. Increasing age and professional experience were linked to stronger role clarity and confidence, reflecting the gradual professional maturation that comes with years of training. Marital status, work schedules, and educational background also seemed to play a part: trainees who held or pursued postgraduate degrees appeared better equipped to manage workplace stressors, whereas those with frequent night shifts expressed higher demands and greater job insecurity. Interestingly, graduates from Greek medical universities reported lower job satisfaction compared with colleagues trained abroad, possibly reflecting enduring structural limitations within national education systems. Overall, these patterns suggest that both personal characteristics and organizational conditions interact to shape residents’ well-being and engagement in the OR [19].

When compared internationally, these associations share similarities with reports for ophthalmology and other surgical specialties in Europe and the United States, where long hours, night shifts, and high emotional demands remain common stressors for surgical trainees [20-23]. Notably, the strong link between opportunities for development and job satisfaction in this sample seems more pronounced than in similar studies, perhaps reflecting the variability in educational experiences within the Greek healthcare system.

From a practical standpoint, reducing excessive quantitative and cognitive demands could be achieved through workload redistribution, optimized surgical scheduling, and improved administrative support [24, 25]. These strategies have been shown to enhance nontechnical skills, decrease cognitive demands, and improve overall surgical performance by reorganizing tasks and providing structured support for residents. Mentoring programs, regular feedback sessions, and conflict resolution training could help strengthen residents’ autonomy, sense of progress, team feeling, and meaning in their work, thereby increasing job satisfaction and team feeling [26-28]. Leadership training for supervising physicians could further improve role clarity, predictability, and overall well-being [29]. Although such interventions are challenging to implement within the resource-limited framework of the Greek public healthcare system, gradual steps, such as introducing structured mentoring in teaching hospitals and improving shift organization, could still be realistic and helpful for enhancing residents’ well-being and training quality.

In conclusion, these findings provide significant contributions to understanding the psychosocial work environment of medical residents in the ORs of Greek public hospitals, with implications for policy and educational practice. Building on these insights, targeted interventions, such as mentoring, leadership development, and workload management, could improve residents’ mental health, workplace relationships, and professional satisfaction. At the same time, addressing job insecurity and clarifying residents’ roles should be prioritized to enhance both well-being and quality of care.

While this study offers meaningful evidence into the psychosocial work environment of ophthalmology residents, some limitations should be considered when interpreting the results. First, its cross-sectional design precludes causal inferences between psychosocial factors and job satisfaction. Second, data collection occurred during the COVID-19 pandemic, which may have affected workload and stress levels. Third, as the study was conducted exclusively in Greek public hospitals, the sole setting for residency training in the country [30], the findings may have limited generalizability to other contexts or healthcare systems. Nevertheless, this unique setting yields valuable insights into an underexplored training environment. Additionally, data on the use of psychiatric medication, alcohol, or substance use, or pre-existing psychological conditions were not collected to protect participant anonymity and encourage honest participation. Finally, all measures were self-reported, introducing the possibility of response and recall bias. Future research involving larger and more diverse samples from multiple institutional settings could further substantiate and extend the present findings.

## Conclusions

This study provides rare national-level evidence on the psychosocial work environment of ophthalmology residents in Greek public hospitals, revealing a clear link between high job demands (quantitative, cognitive, and emotional) and reduced job satisfaction, freedom in work, and team feeling. Conversely, strong job resources such as meaning in work, role clarity, and social support were associated with better well-being and professional fulfillment. These findings underscore the need for systemic reforms in residency training, including structured mentorship, leadership development, optimized workload distribution, and standardized educational frameworks. Addressing job insecurity and clarifying residents’ roles should be central priorities to enhance both workforce well-being and the quality of patient care. Longitudinal and comparative studies are warranted to monitor the impact of these interventions and to align Greek residency programs with international best practices.

## Appendices

**Table 5:** Questionnaire for the assessment of the psychosocial work environment, health, and quality of life The study questionnaire consisted of two parts: 1) demographic and professional characteristics (e.g., age, gender, marital status, education, employment status, and residency training details) and 2) the COPSOQ II COPSOQ II: Copenhagen Psychosocial Questionnaire, second version Source: ​Permission to use the COPSOQ II and its validated Greek version was obtained from the original developers and from the authors of the Greek validation study. The COPSOQ II is licensed under a Creative Commons license (CC BY-NC-ND 4.0; https://www.copsoq-network.org/licence-guidelines-and-questionnaire)

Questionnaire					
When were you are born? 19………. Gender: Male ☐ Female ☐					
Marital status: Free ☐ In relationship ☐ Married ☐ Divorced ☐ Other ☐					
Number of children: ☐					
Working hours: Full-time employment (at least 37.5 hours per week) ☐ Part-time (less than 37.5 hours per week) ☐					
Do you work nights (shifts)? Yes often ☐ Sometimes ☐ No, never ☐					
At which hospital do you work?:					
Speciality					
Position you hold at the specific hospital (e.g., resident, assistant, supervisor, etc.):					
Do you have a master's degree? Yes ☐ No ☐					
Do you hold a PhD? Yes ☐ No ☐					
Which medical school did you attend?					
How many years have you been practicing this specific specialty?					
Are you thinking of changing your major in the future and pursuing another one? Yes ☐ No ☐					
If so, which one will it be?:					
Quantitative requirements	Always	Often	Sometimes	Rarely	Never/almost never
1. Do you have to work at a fast pace?	-	-	-	-	-
2. Is the distribution of your workload uneven, resulting in accumulation of much work?	-	-	-	-	-
3. How often do you not have enough time to finish all your tasks?	-	-	-	-	-
4. Do you fall behind in your work?	-	-	-	-	-
5. How often can you do your work calmly?	-	-	-	-	-
6. Do you have enough time to finish your work?	-	-	-	-	-
Cognitive requirements	Always	Often	Sometimes	Rarely	Never/almost never
7. Do you have to pay attention to many things simultaneously during your work?	-	-	-	-	-
8. Does your job require you to have a very good memory?	-	-	-	-	-
9. Does your job require you to be creative?	-	-	-	-	-
10. Do you have to make difficult decisions in your job?	-	-	-	-	-
Emotional demands	To a very large extent	To a large extent	To a moderate extent	To a small extent	To a very small extent
11. Does your work create emotionally disturbing situations for you?	-	-	-	-	-
12. Are there emotional demands in your work?	-	-	-	-	-
13. Do you become emotional at work?	-	-	-	-	-
Requirements to cover emotions	-	-	-	-	-
14. Does your work require you to hide your emotions?	-	-	-	-	-
15. Are you forbidden to express your opinion at work?	-	-	-	-	-
Sensory demands	Always	Often	Sometimes	Rarely	Never/almost never
16. Does your work require very good vision?	-	-	-	-	-
17. Is it required to control your movements consciously (e.g., hands and fingers)?	-	-	-	-	-
18. Do you have to be constantly attentive?	-	-	-	-	-
19. Does your work require a high degree of precision?	-	-	-	-	-
Influence at work	Always	Often	Sometimes	Rarely	Never/ almost never
20. Do you have a large influence on your work?	-	-	-	-	-
21. Can you influence the amount of work you are assigned?	-	-	-	-	-
22. Do you have any influence over what you do at work?	-	-	-	-	-
Possibilities for development	To a very large extent	To a large extent	To a moderate extent	To a small extent	To a very small extent
23. Does your job require you to take initiative?	-	-	-	-	-
24. Does your type of work vary?	-	-	-	-	-
25. Do you have the opportunity to learn new things through your work?	-	-	-	-	-
26. Do you allow yourself to use your skills and experience in your job?	-	-	-	-	-
Degree of freedom at work	Always	Often	Sometimes	Rarely	Never/almost never
27. Can you decide for yourself when to take breaks?	-	-	-	-	-
28. Do you take your leave, more or less, whenever you want?	-	-	-	-	-
29. Are you able to interrupt your work to chat with a colleague?	-	-	-	-	-
30. If a personal issue arises, are you able to take half an hour off work without special permission?	-	-	-	-	-
Meaning in work	To a very large extent	To a large extent	To a large extent	To a small extent	To a very small extent
31. Do you find meaning in your work?	-	-	-	-	-
32. Do you feel that the work you do is important?	-	-	-	-	-
33. Do you feel motivated by your work?	-	-	-	-	-
Commitment to the working environment	To a very large extent	To a large extent	To a large extent	To a small extent	To a very small extent
34. Would you like to stay in your current work environment for the rest of your working life?	-	-	-	-	-
35. Do you enjoy telling other people about your workplace?	-	-	-	-	-
36. Do you feel like problems at work are also your problems?	-	-	-	-	-
37. Do you feel that the position you work in is of great importance to you?	-	-	-	-	-
Predictability	To a very large extent	To a large extent	To a moderate extent	To a small extent	To a very small extent
38. Are you informed in a timely manner about important decisions, changes, or future plans in your workplace?	-	-	-	-	-
39. Do you receive all the information you need to do your work well?	-	-	-	-	-
Clarity in roles					
40. Do you know exactly how much your opinion counts at work?	-	-	-	-	-
41. Does your job have clear goals?	-	-	-	-	-
42. Do you know exactly what your responsibilities are?	-	-	-	-	-
43. Do you know exactly what is expected of you at work?	-	-	-	-	-
Role conflict	-	-	-	-	-
44. Do you do things in your work that are considered acceptable by some but not by others?	-	-	-	-	-
45. Are there contradictory demands at your work?	-	-	-	-	-
46. Are you sometimes forced to carry out your work in an unorthodox way?	-	-	-	-	-
47. Are you sometimes forced to do things that seem unnecessary?	-	-	-	-	-
Quality of leadership					
48. Is your supervisor good at organizing the work?	-	-	-	-	-
49. Is your supervisor good at resolving conflicts?	-	-	-	-	-
50. Does your supervisor make sure each employee has opportunities for development that they deserve?	-	-	-	-	-
51. Does your supervisor place great importance on the satisfaction the employee can get from their work?	-	-	-	-	-
Social support	Always	Often	Sometimes	Rarely	Never/almost never
52. How often do you receive help and support from your colleagues?	-	-	-	-	-
53. How often are your colleagues willing to listen to work-related problems?	-	-	-	-	-
54. How often is your immediate superior willing to listen to your work-related problems?	-	-	-	-	-
55. How often do you receive help and support from your supervisor?	-	-	-	-	-
Support at work	Always	Often	Sometimes	Rarely	Never/almost never
56. How often does your supervisor tell you how well you are doing your work?	-	-	-	-	-
57. How often do your colleagues tell you how well you are doing in your work?	-	-	-	-	-
Social relations					
58. Do you work isolated from your colleagues?	-	-	-	-	-
59. Do you have the possibility to talk with your colleagues while working?	-	-	-	-	-
Team feeling					
60. Is there a good atmosphere among you and your colleagues?	-	-	-	-	-
61. Is there good cooperation between colleagues at your workplace?	-	-	-	-	-
62. Do you feel part of a team at your work?	-	-	-	-	-
Jobs insurance	Yes	No			
63. Are you worried about becoming unemployed?	-	-			
64. Is new technology making you feel useless?	-	-			
65. Would you find it difficult to find a new job if you became unemployed?	-	-			
66. Have you been transferred to another department against your will?	-	-			
Job satisfaction (Regarding your job in general, how satisfied are you with)	Very satisfied	Satisfied	Dissatisfied	Very dissatisfied	Not by any means
67. Your job prospects?	-	-	-	-	-
68. The physical working conditions?	-	-	-	-	-
69. The way your skills are used?	-	-	-	-	-
70. With your work as a whole, including everyone?	-	-	-	-	-
General health status	Excellent	Very good	good	A little good	Bad
71. In general, would you say that your health is	-	-	-	-	-
Mark whether each of the following statements is true or false	Absolutely true	Usually true	I don't know	Usually wrong	Absolutely wrong
72. I get sick more easily than other people	-	-	-	-	-
73. I am as healthy as anyone else	-	-	-	-	-
74. I believe my health condition will get worse	-	-	-	-	-
75. My health is excellent	-	-	-	-	-
Mental health (These are questions about how you have been feeling and how things have been going over the past 4 weeks. For each question, please give the answer that comes closest to how you have been feeling. For how long during the past 4 weeks)	All the time	Most of the time	Much of the time	A few times	Never
76. Were you very nervous?	-	-	-	-	-
77. You felt so down that nothing could lift you up?	-	-	-	-	-
78. Did you feel calm?	-	-	-	-	-
79. Did you feel disappointed and melancholic?	-	-	-	-	-
80. Did you feel like a "happy person"?	-	-	-	-	-
Vitality (These are questions about how you have been feeling and how things have been going over the past 4 weeks. For each question, please give the answer that comes closest to how you have been feeling. For how long during the past 4 weeks)	All the time	Most of the time	Much of the time	A few times	Never
81. Did you feel surrounded by shoulds?					
82. Did you have a lot of energy?					
83. Did you feel exhausted?					
84. Did you feel tired?					
Behavioral stress (Please mark which description best fits your situation during the last 4 weeks)	Correct	Almost Correct	About Correct	A little dose of truth	Wrong
85. I couldn't face other people					
86. I didn't have time to relax and enjoy myself					
87. I was a little irritable					
88. I didn't take any initiatives					
Physical stress (During the last 4 weeks, how many times have you)	Always	Often	Sometimes	Rarely	Never/almost never
89. Have you had a stomach ache or stomach discomfort?	-	-	-	-	-
90. Have you felt chest tightness or chest pain?	-	-	-	-	-
91. Did you feel dizzy?	-	-	-	-	-
92. Did you feel tightness in your muscles?	-	-	-	-	-
Cognitive stress (During the past 4 weeks, how many times have you)	Always	Often	Sometimes	Rarely	Never/almost never
93. Have you had problems concentrating?	-	-	-	-	-
94. Have you had difficulty making decisions?	-	-	-	-	-
95. Have you had difficulties with your memory?	-	-	-	-	-
96. Have you had trouble thinking clearly?	-	-	-	-	-
